# Determine Neuronal Tuning Curves by Exploring Optimum Firing Rate Distribution for Information Efficiency

**DOI:** 10.3389/fncom.2017.00010

**Published:** 2017-02-21

**Authors:** Fang Han, Zhijie Wang, Hong Fan

**Affiliations:** ^1^Department of Automation, College of Information Science and Technology, Donghua UniversityShanghai, China; ^2^Center for Neural Science, New York UniversityNew York, NY, USA; ^3^Department of Information Management System, Glorious Sun School of Business and Management, Donghua UniversityShanghai, China

**Keywords:** neuronal tuning curve, information efficiency, optimum firing rate distribution, mutual information, energy consumption

## Abstract

This paper proposed a new method to determine the neuronal tuning curves for maximum information efficiency by computing the optimum firing rate distribution. Firstly, we proposed a general definition for the information efficiency, which is relevant to mutual information and neuronal energy consumption. The energy consumption is composed of two parts: neuronal basic energy consumption and neuronal spike emission energy consumption. A parameter to model the relative importance of energy consumption is introduced in the definition of the information efficiency. Then, we designed a combination of exponential functions to describe the optimum firing rate distribution based on the analysis of the dependency of the mutual information and the energy consumption on the shape of the functions of the firing rate distributions. Furthermore, we developed a rapid algorithm to search the parameter values of the optimum firing rate distribution function. Finally, we found with the rapid algorithm that a combination of two different exponential functions with two free parameters can describe the optimum firing rate distribution accurately. We also found that if the energy consumption is relatively unimportant (important) compared to the mutual information or the neuronal basic energy consumption is relatively large (small), the curve of the optimum firing rate distribution will be relatively flat (steep), and the corresponding optimum tuning curve exhibits a form of sigmoid if the stimuli distribution is normal.

## Introduction

Neuronal systems are assumed to be optimized for information encoding after millions of years of natural selection, in the sense that the capacity of the neural systems (or channels in the language of information society) for information transmission is occupied as much as possible, leaving as little as possible the “power” of the channels being unutilized. This is the “redundancy reduction” hypothesis proposed by Barlow (1959, 1961). Based on this assumption, the optimum models of the neural systems are obtained using information theory (Atick and Redlich, 1990; Borst and Theunissen, 1999; Bethge et al., 2003; Nikitin et al., 2009; McDonnell et al., 2011; Rolls and Treves, 2011; Wei and Stocker, 2016). These optimum models coincide with the existing results well, implying that the way how neural systems process information can be understood and the models of the neural systems can be constructed by the information theory.

As energy consumption of the brain occupies a large part of the total energy consumption of organisms (Erecinska et al., 2004), some studies optimized neural systems by maximizing the information efficiency, i.e., maximizing the ratio of the mutual information to the energy consumed by the neuron for emitting spikes (Levy and Baxter, 1996; Wang and Zhang, 2007; Torreal dea Francisco et al., 2009; Berger and Levy, 2010; Sengupta and Stemmler, 2014; Sengupta et al., 2014). Some studies (Levy and Baxter, 1996; Attwell and Laughlin, 2001) further elaborated the definition of the information efficiency by taking account of two parts of energy consumption of a neuron. Besides the part of the energy consumed by the neurons to fire spikes (Yu and Liu, 2014), they considered another part of energy consumption, called basic energy consumption, which is relevant to the metabolic cost required to support the living of the neurons or the sub-threshold activity of the neurons. Evidently, the first part of energy consumption is proportional to the number of the spikes, while the second part of the energy consumption has no relationship with the number of the spikes.

Tuning curve describes the relationship between the stimulus and the output of the neuron, which plays a very important role in neuronal information encoding (McDonnell and Stocks, 2008; Nikitin et al., 2009; Yaeli and Meir, 2010; Day et al., 2012; Wang et al., 2013; Han et al., 2015; Yarrow and Series, 2015). Tuning curve is optimized for information encoding to test the “redundancy reduction” hypothesis at the single neuron level (Laughlin, 1981). Considering the energy consumption, tuning curves should be optimized to enable the neurons to encode information efficiently, i.e., to have high ratio of information to energy consumption. To obtain tuning curves in this way, the following three elements need to be considered: the calculation of energy consumption (Levy and Baxter, 1996; Wang and Zhang, 2007; Torreal dea Francisco et al., 2009; Berger and Levy, 2010; Sengupta and Stemmler, 2014; Sengupta et al., 2014), the definition of the information efficiency (Levy and Baxter, 1996; Moujahid et al., 2011; Kostal and Lansky, 2013; Sengupta et al., 2014), and the probability distribution of inputs (Dayan and Abbott, 2001; Nikitin et al., 2009). Most of the existing methods for determining optimum tuning curves assumed simplified situations of above-mentioned three elements, especially the first two. In some methods, energy consumption was not taken into account. For example, early work on the optimization of the tuning curve (Laughlin, 1981) by entropy maximization validates the “redundancy reduction” hypothesis; a general method for determining tuning curves for maximizing mutual information was proposed in McDonnell and Stocks (2008); the optimum tuning curve for maximizing mutual information was found to have a discrete structure (Nikitin et al., 2009); the optimal tuning functions for minimum mean square reconstruction from neural rate responses were derived in Bethge et al. (2003). In other methods the number of spikes was regarded as the energy consumption, and the ratio of the mutual information to the number of the spikes was used as the information efficiency (Moujahid et al., 2011; Kostal and Lansky, 2013). However, in real neural systems, things seem to be more complex. For example, two parts of the energy consumption rather than only spikes should be considered as discussed in the previous paragraph. As for the definition of the information efficiency, one should consider the fact that in some situations, mutual information is more important than energy consumption, while in some other cases, energy consumption should be underlined. The information and the energy consumption should also be measured in the same scale when calculating information efficiency, despite that information is measured on logarithm scale while energy on linear scale in most existing studies. Furthermore, the probability distribution of the inputs in many cases may take a complex form other than normal distribution. How can we determine the neuronal tuning curves with these complex situations considered?

It is usually difficult to obtain analytical solutions to the tuning curves when the complex situations discussed in the previous paragraph are considered. This paper proposes a new computer algorithm to deal with these complex situations when optimizing neuronal tuning curves. Firstly, the optimum spike count response distribution (the probability distribution of the numbers of the spikes emitted by the neuron for different inputs, which is explained in details in Section The model for information-efficiency in neuronal encoding system) is analyzed in terms of full entropy and energy consumption, and a combination of exponential-based functions is designed for it. Then the optimum firing rate distribution (see the detailed explanations in Section The model for information-efficiency in neuronal encoding system) for the information efficiency is explored. Based on the analysis of the relationship between the optimum spike count response distribution and the optimum firing rate distribution, a combination of exponential-based functions is designed for the optimum firing rate distribution, after the dependency of the noise entropy on the shape of the functions of the firing rate distribution is analyzed. A forward–backward rapid algorithm with variable step size is proposed for searching the optimum neuronal firing rate distribution function. It is found with the rapid algorithm that a combination of two different exponential functions with two free parameters can describe the optimum firing rate distribution accurately. The rapid algorithm is then used to search the two parameter values of the optimum neuronal firing rate distribution function. Finally, the neuronal tuning curves are calculated based on the optimum neuronal firing rate distribution and the stimuli probability distribution. The paper is organized as follows. The basic concept of the optimum neuronal encoding scheme is described in Section The model for information-efficiency in neuronal encoding system. A new method to search the optimum neuronal firing rate distribution is proposed in Section Search for the optimum neuronal firing rate distribution. The optimum tuning curves are calculated based on the optimum neuronal firing rate distribution in Section The tuning curves based on the optimum neuronal firing rate distribution. Finally, a brief summary is provided in Section Conclusions.

## The model for information-efficiency in neuronal encoding system

A neuron receives inputs from the environment and encodes them in the spikes of the neuron. For each input *s*, the neuron responses with *n* spikes within time window *T* (we suppose that neurons must complete the encoding process within this short time period to get a rapid response to the external stimulus) (Bethge et al., 2003). Since neuronal responses are naturally random, the value of *n* is different trial by trial even the same input *s* is provided, i.e., *n* is a random variable, which is often assumed to obey Poisson distribution for simplification (Dayan and Abbott, 2001; Bethge et al., 2003; Nikitin et al., 2009). The rate of this Poisson distribution is called the firing rate of the neuron. Let *r* = *g*(*s*) be the tuning curve of the neuron, which we want to determine according to information theory in this paper. Suppose the input *s* appears with probability *P*(*s*), which can often be measured by recording the frequency of the occurrence of the stimulus *s* (The frequency of the occurrence of the stimulus between *s* and *s* + Δ*s* is recorded for *P*(*s*) if the stimulus is continuous). If we know the probability density of the firing rate *p*(*r*), i.e., the firing rate distribution, then we can calculate the tuning curve, *r* = *g*(*s*), numerically (see the details in Section The tuning curves based on the optimum neuronal firing rate distribution). How can we get the firing rate distribution *p*(*r*)? We now discuss this according to the assumption that a neuron is an optimum encoding system (In some situations, the spike sequence emitted by the neuron could be recorded and used to calculate *p*(*r*). This *p*(*r*) could be used to validate the redundancy reduction hypothesis by comparing it with the *p*(*r*) that is got by information efficiency maximization).

As we discussed in the previous section, the assumption that a neuron is an optimum encoding system means that the neuron can convey a large amount of information on one hand, while consume a small amount of energy on the other hand. Stated in other words, the neuron exhibits optimum information encoding efficiency. The amount of information conveyed by the neuron (mutual information) (Kostal, 2010; Gao et al., 2014) can be described by *I*_*m*_ = *S*_*full*_ − *S*_*noise*_, where *S*_*full*_ is the full entropy of the neuronal response (see Equation 2) and *S*_*noise*_ (see Equation 3) is the noise entropy. As for the energy consumption, the basic energy consumption of the neuron within the period *T* is *E*_*b*_ and the energy consumed by the neuron to emit spikes is *E*_*s*_, with total energy consumption *E* = *E*_*b*_ + *E*_*s*_. We measure the energy consumption in units of number of spikes. For example, *E*_*b*_ = 2 means that the basic metabolic cost required to support the living of the neuron within *T* is equivalent to the energy consumed by the neuron to emit 2 spikes.

A simple but widely used definition of the information efficiency, *I*_*E*_, is the ratio of mutual information to the energy consumed by neurons to emit spikes, i.e., $I_{E}=\frac{I_{m}}{E_{s}}$ (Levy and Baxter, 1996; Moujahid et al., 2011; Sengupta and Stemmler, 2014). This definition is extended in this paper, which is described by Equation (1).

(1) $$\begin{array}{r} I_{E}=\left(2^{I_{m}}-1\right)/{\left(E_{s}+E_{b}\right)}^{c} \end{array}$$

This extension is motivated by the following three considerations. The first is that energy consumption is composed of two parts in real neurons as discussed previously. Thereby *E* = *E*_*s*_ + *E*_*b*_ (Levy and Baxter, 1996; Attwell and Laughlin, 2001) instead of *E*_*s*_ is used in the calculation of *I*_*E*_. The second is that in some situations, mutual information (energy consumption) may be considered more important than energy consumption (mutual information). The exponential *c* in Equation (1) is used to model this relative importance in these situations. When *c* < 1, mutual information is considered more important; when *c* > 1, energy consumption is more focused on. The third is that mutual information is measured in bits, therefore it should be transformed into linear representation as energy consumption. That's why we use $2^{I_{m}}-1$ for calculation of *I*_*E*_.

Suppose the inputs are discretized as s(*i*) = *i*Δ*s*, where *i* = 1,2…*M*, Δ*s* = 0.0001 and *M* = 10,000 (the stimuli are normalized in this paper). The probability of the occurrence of *s*(*i*) is *P*(*s*(*i*)) = (*p*(*s*)|*s*(*i*))Δ*s*. The firing rates of neurons are discretized as *r*(*i*) = *r*(i − 1) + Δ*r, i* = 1,2…*N*, Δ*r* = 0.1 and *N* = 5, 000. The probability of the occurrence of *r*(*i*) is *P*(r(*i*)) = (*p*(*r*)|*r*(*i*))Δ*r*. Suppose the probability that the neuron emits *n* spikes within time window *T* is *P*(*n*) (we name *P*(*n*) the spike count response distribution in this paper), then the full entropy of the neuron response is

(2) $$\begin{array}{r} S_{full}=\sum_{n}P(n)log_{2}\;P(n), \end{array}$$

and the noise entropy of the firing rate of the neuron is

(3) $$\begin{array}{l} S_{noise}=\sum_{j}\sum_{n}P(s(j))P(n|s(j))log_{2}\;P(n|s(j))\\ \;=\sum_{j}\sum_{j}P(r(j))P(n|r(j))log_{2}(P(n|r(j)), \end{array}$$

where *P*(*n*|*s*(*j*)) is the conditional probability and *r*(*j*) = *g*(*s*(*j*)).

## Search for the optimum neuronal firing rate distribution

If the optimum firing rate distribution for information efficiency and the input distribution are given, one can calculate the tuning curve numerically (it will be discussed in Section The tuning curves based on the optimum neuronal firing rate distribution). In this section, we will discuss how to determine the optimum firing rate distribution for information efficiency. To achieve this, we first explore the optimum spike count response distribution for the entropy efficiency (entropy efficiency considers full entropy and energy consumption) in Section Optimum spike count response distribution for entropy efficiency. In Section Optimum neuronal firing rate distribution for entropy efficiency, we determine optimum neuronal firing rate distribution for the entropy efficiency based on the assumption that the number of the firing of the neuron obeys Poisson distribution when firing rate is fixed. We find using the non-linear least square method that the optimum firing rate distribution for the entropy efficiency has the same form of function as the optimum spike count response distribution but having different parameter values. In Section Optimum neuronal firing rate distribution for information efficiency, to extend the entropy efficiency to information efficiency, the noise entropy is included in the framework (note that entropy efficiency considers full entropy but information efficiency considers mutual information) and we found that the form of the firing rate distribution function for the maximum information efficiency is the same as that for entropy efficiency. Finally, in Sections Algorithm for searching the optimum neuronal firing rate distribution and Searching the optimum neuronal firing rate distribution, a rapid algorithm is proposed to calculate the optimum firing rate distributions.

### Optimum spike count response distribution for entropy efficiency

Let us begin with a simple case, entropy efficiency, which only considers full entropy of the spike count neuronal response and energy consumption. Similar to the information efficiency, we define entropy efficiency, *IS*, as $IS=\left(2^{S_{full}}-1\right)/{\left(E_{s}+E_{b}\right)}^{c}$. As we know, the neuronal response should be uniformly distributed for the maximum entropy, i.e., *P*(*n*) = 1/*n*_*max*_ where *n*_*max*_ is the maximum spike number of the neuron within the measuring time window *T*. But maximum entropy does not mean maximum entropy efficiency, because the value of energy consumption is relatively large if *P*(*n*) = 1/*n*_*max*_ because $E_{s}=\sum_{n}P\left(n\right)n$. As $\frac{\text{d}E_{s}}{dP\left(n\right)}=n$, to reduce energy consumption, the function *f*(*n*) = *P*(*n*) should be smaller with larger *n*. Namely, to decrease the energy consumption, the function *f*(*n*) should decrease hardly to 0, i.e., *f*(*n*) should decrease monotonously, and the rate of the decrease should also decrease with the increase of *n*. Thereby we can conceive that exponential-like function can describe such functions that lead to small energy consumption and large entropy. Since $\sum_{n}f(n)=1$, the simplest function of *f*(*n*) is

(4) $$\begin{array}{r} f\left(n\right)=\alpha e^{-\alpha n}. \end{array}$$

**Figure 1:**
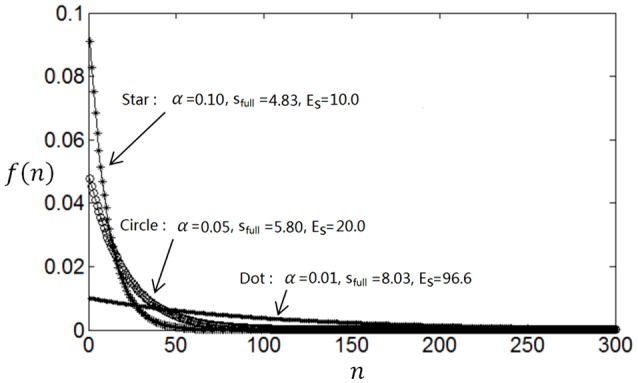
**Dependency of full entropy and energy consumption on the parameter α**.

The entropy will be smaller when *f*(*n*) deviates more from uniform distribution. Therefore, if the parameter α is large then *f*(*n*) will decay rapidly, which will result in less energy consumption (*E*_*s*_) but small full entropy (see Figure 1); otherwise if α is small, then *f*(*n*) will decay slowly, which will result in large energy consumption (*E*_*s*_) and large full entropy (see Figure 1). If α is very small, *f*(*n*) will be almost flat leading to maximum full entropy. Therefore, both energy consumption and full entropy decrease with the increase of α, but the rates of the decrease are different, which enables the optimum value of α leading to maximum information efficiency (see Section Algorithm for searching the optimum neuronal firing rate distribution for details).

The functions *f*(*n*) = α*e*^−α*n*^ have only one free parameter α. This restrains the possible shapes of the spike count response distribution functions. To make the possible shapes of the distribution functions more various, we use a function that is a combination of two exponential functions with two free parameters as Equation (5).

(5) $$\begin{array}{r} f\left(n\right)=\left(\alpha e^{-\alpha n}+\beta e^{-\beta n}\right)/2. \end{array}$$

We can also use a function with three free parameters as Equations (6) or (7).

(6) $$\begin{array}{r} f\left(n\right)=\left(\alpha e^{-\alpha n}+\beta e^{-\beta n}+\gamma e^{-\gamma n}\right)/3, \end{array}$$

(7) $$\begin{array}{r} f\left(n\right)=\alpha e^{-\alpha n}\gamma +\beta e^{-\beta n}\left(1-\gamma \right). \end{array}$$

Note that *n* is an integer, the values of γ in Equation (7) lie between 0 and 1, and for all these functions, $\sum_{n}f(n)=1$. We design the function as such a form that both full entropy and *E*_*s*_ decrease monotonically with the increase of either α or β. This allows us to design a rapid algorithm (see Section Algorithm for searching the optimum neuronal firing rate distribution) to search the optimum parameters.

### Optimum neuronal firing rate distribution for entropy efficiency

If the response of the neuron is non-random, then average spike number over multiple observations (firing rate) is identical to the spike number measured in a single observation. Thereby, firing rate distribution is identical to the spike count response distribution. However, neuronal response is random, which usually follows Poisson distribution (Dayan and Abbott, 2001; Bethge et al., 2003; Nikitin et al., 2009). It is interesting to see that the function of firing rate distribution takes the same form as that of spike count response distribution but with different parameters, when this randomness is taken into account. That is, if we use a two-free-parameter function, the function of firing rate distribution may be described by Equation (8) [note that unlike the neuronal response function of Equation (5) where *n* is an integer, *r* is a continuous variable in Equation (8)]:

(8) $$\begin{array}{r} f\left(r\right)=\left(\alpha e^{-\alpha r}+\beta e^{-\beta r}\right)/2. \end{array}$$

**Figure 2:**
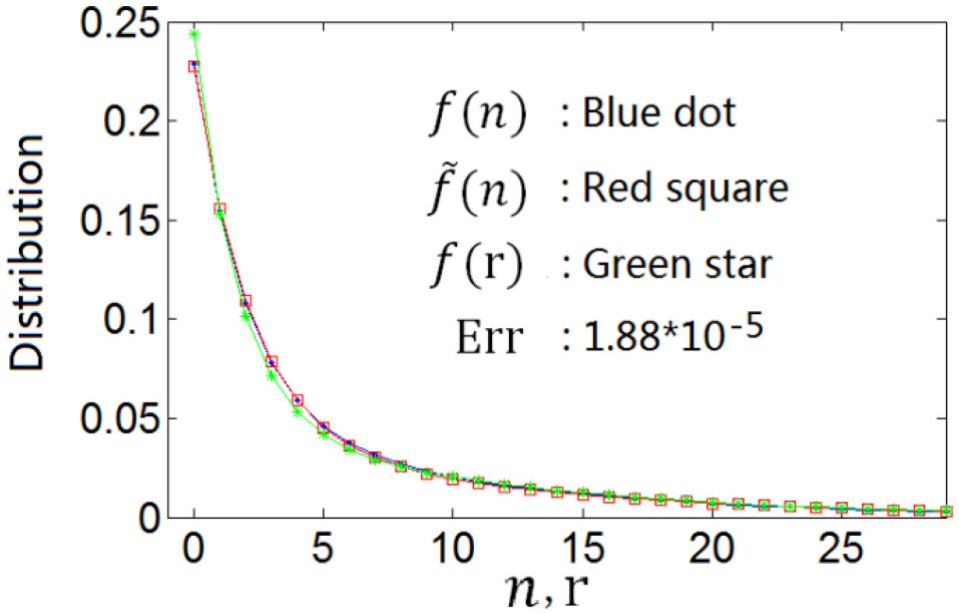
**The optimum spike count response distribution (blue dot) described by Equation (5) and the firing rate distribution (green star) searched by the nonlinear least square method which produces the spike count response distribution (red square) fitting the optimum spike count response distribution perfectly**.

This can be confirmed by the nonlinear least square method. For example, the curve with blue dot in Figure 2 is the spike count response distribution produced by Equation (5) with parameters α = 0.1 and β = 0.5. We denote this curve as *f*(*n*). We search the best firing rate distribution denoted as *f*(*r*), which can produce a spike count response distribution fitting the blue dot curve (*f*(*n*)) most perfectly using the nonlinear least square method. We got a function of Equation (8) with α = 0.0935 and β = 0.5882 for the firing rate distribution, which is shown by the curve with green star in Figure 2. The curve with red square, which we denote as $\tilde{f}\left(n\right)$, is the spike count response distribution that is produced by this firing rate distribution (note that if the firing rate *r* is fixed, the neuronal spike count response *n* obeys Poisson distribution). Red square curve ($\tilde{f}\left(n\right)$) fits blue dot curve (*f*(*n*)) perfectly, implying that the optimum firing rate distribution for the entropy efficiency can also be described by the continuous version of Equation (5), i.e., Equation (8). We use $Err=\sum_{n}{\left(f\left(n\right)-\tilde{f}\left(n\right)\right)}^{2}$ to characterize the distance of the two neuronal spike count response functions. It is shown from *Err* = 1.88 × 10^−5^ for the red square curve and blue dot curve that the two curves are almost identical. More experimental results are shown in Table 1. The first line of Table 1 shows *f*(*n*) described by Equation (5) with various parameter values of α and β; the second line shows *f*(*r*) which is described by the continuous version of Equation (5) (Equation 8) but having different parameter values of α and β; and the third line shows *Err* (note that *Err* is not the distance between *f*(*n*) and *f*(*r*), instead it is the distance between *f*(*n*) and $\tilde{f}\left(n\right)$). The results in Table 1 confirm that if the optimum neuronal spike count response can be described by Equation (5), the optimum firing rate can also be described by continuous version of Equation (5) (Equation 8).

**Table 1:** *****f***(***n***) with various values of (α, β), ***f***(***r***) with values of (α, β) and ***Err*****.

*f*(*n*)	(0.10, 0.50)	(0.20, 0.50	(0.30, 0.50)	(0.40, 0.50)	(0.20, 0.60)	(0.30, 0.80)	(0.30, 0.90)
*f*(*r*)	(0.09, 0.59)	(0.21, 0.62)	(0.34, 0.64)	(0.49, 0.65)	(0.21, 0.76)	(0.32, 1.12)	(0.31, 1.27)
*Err*	1.9 × 10^−5^	8.6 × 10^−7^	1.1 × 10^−7^	8.5 × 10^−8^	3.9 × 10^−6^	4.4 × 10^−6^	1.1 × 10^−5^

### Optimum neuronal firing rate distribution for information efficiency

Since mutual information of the neuronal response is the actual amount of information encoded in the output of the neuron, it is more important to discuss information efficiency than entropy efficiency. Based on the entropy efficiency discussed in the previous two subsections, we consider the noise entropy in the following study and discuss the optimum firing rate distribution for information efficiency, i.e., determine the form of the function of the neuronal firing rate distribution that results in more mutual information but less energy consumption. Let us first consider what kind of neuronal firing rate distribution can lead to less noise entropy. We first divide the noise entropy *S*_*noise*_ into components with each component associated with individual firing rate *r*. According to Equation (3), $S_{noise}=\sum_{j}\sum_{n}P(r(j))P(n|r(j))log_{2}\;P(n|r(j))$. Let

(9) $$\begin{array}{r} S_{noise}=\sum_{j}P(r(j))N(r), \end{array}$$

where *N*(*r*) represents the increase rate of the noise entropy to the probability *P*(*r*(*j*)), we have

(10) $$\begin{array}{r} N(r)=\sum_{n}P(n|r(j))\;log_{2}P(n|r(j)), \end{array}$$

where *P*(*n*|*r*(*j*)) is the possibility that the neuron emits *n* spikes when the firing rate of the neuron is *r*(*j*). *P*(*n*|*r*(*j*)) follows Poisson distribution as *P*(*n*|*r*(*j*)) = *e*^−*r*(*j*)^*r*(*j*)^*n*^/*n*!.

**Figure 3:**
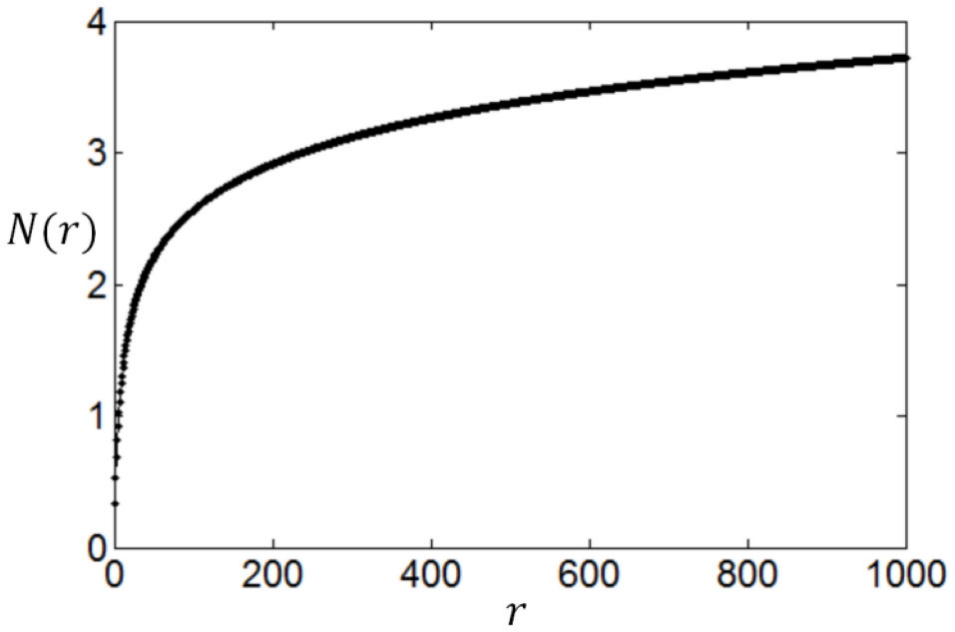
**The dependency of increase rate of noise entropy on the firing rate**.

We compute *N*(*r*) with different *r* and plot the dependency of *N*(*r*) on *r* in Figure 3. It can be seen from Figure 3 that the noise entropy component *N*(*r*) increases with *r*. Therefore, we can infer according to Equation (9) and Figure 3 that if the value of *P*(*r*) decreases with the increase of *r* then *S*_*noise*_ will be small, very similar to the fact that if the value of *P*(*r*) decreases with the increase of *r* then energy consumption will be small. Thus, we conclude that a firing rate distribution function capable of producing high full entropy, low noise entropy and less energy consumption should have a shape like that in Figure 1 (or **Figures 7**–**9**). Namely, we can use the functions of Equations (4)–(7) to describe the optimum firing rate distribution function for the maximum information efficiency.

It is worthy of noting that by treating the noise entropy in this way, we can see the effect of the noise entropy on the shape of the optimum firing rate distribution clearly, thereby we can extend the application of our method to other neural response models. Our method applies for any neural response models as long as *N*(*r*) has a shape like Figure 3, i.e., *N*(*r*) increases rapidly and then slowly with the increase of *r*. For example, we found that a Poisson process with a stochastic refractory period (Bair et al., 1994) and a negative binomial distribution (Goris et al., 2014) produce *N*(*r*) that have similar shape as Figure 3. Thereby our method is also applicable to these two neural response models.

### Algorithm for searching the optimum neuronal firing rate distribution

We first give a short summary of our discussion in previous sections. Our idea is to determine the shape of the optimum firing rate distribution by heuristically analyzing the effect of the full entropy, energy consumption and noise entropy on the shape of the optimum firing rate distribution. As it is relatively easier to observe the effect of the full entropy and energy consumption on the shape of the optimum spike count response distribution, we first determine the shape of optimum spike count response distribution with only full entropy and energy consumption taken into account and use a combination of exponential functions to describe the optimum spike count response distribution. Then we confirm by using the nonlinear least square method that the optimum spike count response distribution described by the combination of exponential functions can be produced by a firing rate distribution which can also be described by a combination of exponential functions. We further discuss the effect of the noise entropy on the shape of the optimum firing rate distribution by calculating *N*(*r*) described in Section Optimum neuronal firing rate distribution for information efficiency. Thus, the shape of the optimum of the firing rate distribution is determined. It is interesting to see by numerical calculation in Section Searching the optimum neuronal firing rate distribution that the combination function of a pair of exponents can describe the optimum firing rate distribution well enough when Poisson process is adopted, therefore we use a family of low-parametric functions (combination functions of a pair of exponents) in this paper. Of course, the combination functions of two exponents may not be good enough when other neuronal response models are adopted. Three exponents or other kinds of functions may need to be used to describe the optimum firing rate distribution in other cases.

**Figure 4:**
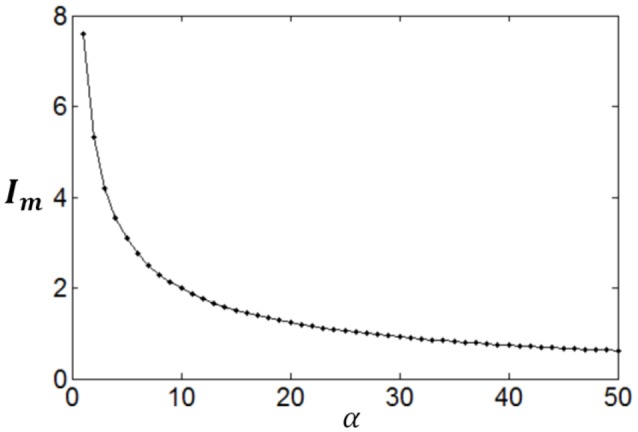
**The change of mutual information with the parameter α**.

**Figure 5:**
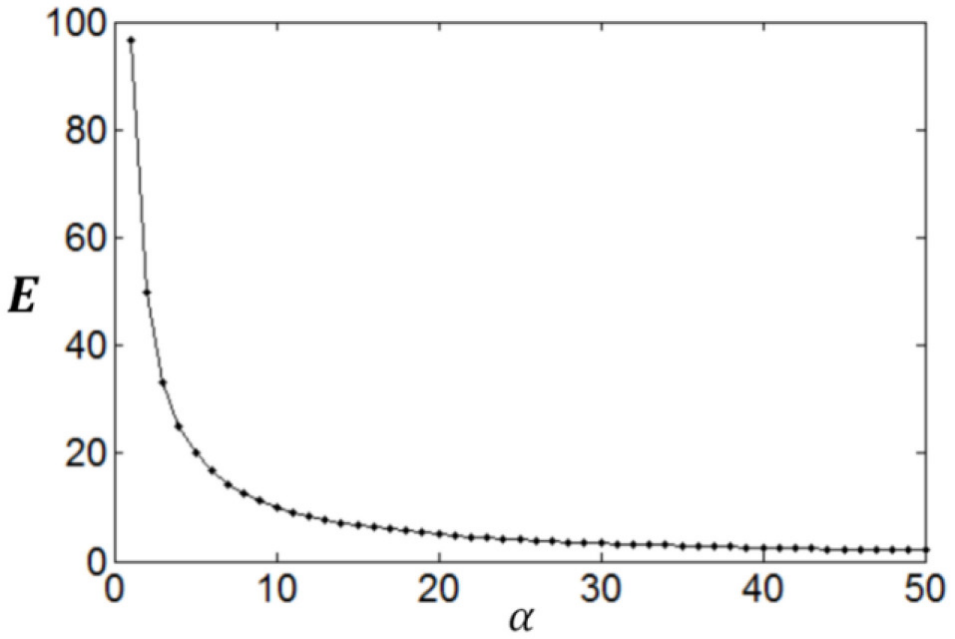
**The change of energy consumption with the parameter α**.

Poisson process is adopted for the neuron response, therefore optimum firing rate distribution can be described by the functions of Equations (4)–(7). It is worthy of noting that although a family of low-parametric functions is used in the paper, it is difficult to solve the problem analytically. This is because the object to be optimized (maximized) is Equation (1), i.e., the optimum firing rate distribution is searched to achieve both high exponential-weighted information and low power-weighted energy consumption concurrently. This optimization problem is different from that of mutual information maximization when energy consumption is fixed. Therefore, we next discuss how to design algorithms to search the parameter values of the optimum firing rate distribution functions for the maximum information efficiency in this subsection. Firstly, let us consider the simplest neuronal firing rate distribution function with only one parameter α. If α gets larger, *p*(*r*) decreases more sharply with the increase of *r*, resulting in a smaller amount of energy consumption, full entropy [note that the flatter of the curve of *p*(*r*), the larger the full entropy], and noise entropy [note that noise entropy is small if *p*(*r*) is small for large values of *r* according to Figure 3]. Figures 4, 5 show that the values of mutual information and energy consumption decrease monotonically with increasing the parameter α. The larger α gets, more slowly these quantities decrease with α. Finally, they approach to their limits, respectively. Therefore, we can design a variable step size scheme to search the optimum parameters. Specifically, we can use small step sizes when α is small, but use relatively large step sizes when α is large. In this paper, we let α(*i*) = α(*i* − 1) + α(*i* − 1)/log_2_(200 × α(*i* − 1)) and α(1) = 0.01.

**Figure 6:**
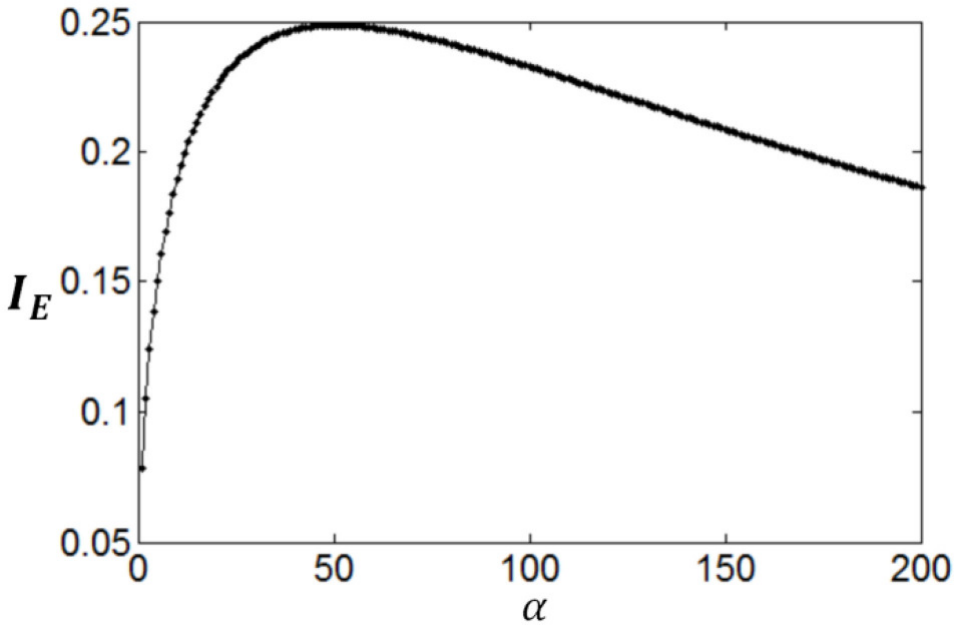
**The change of information efficiency with the parameter α; ***c*** = 1 and ***E***_***b***_ = 1**.

Furthermore, it can be seen that although both mutual information and energy consumption decrease with the increase of α, the decrease rates are different. This is shown in Figure 6. When α is small, the decrease rate of energy consumption is more rapid, meaning that information efficiency increases with the increase of α. With the increase of α, the difference between the decrease rates of energy consumption and mutual information becomes smaller and smaller, finally vanishes. Accordingly, the increase rate of information efficiency becomes smaller and smaller, and finally reaches the maximum value as shown in Figure 6. To search this maximum point and the corresponding parameter α, we develop a forward–backward algorithm with forward phase and backward phase described as follows.

Forward phase: starting from the initial point α(*i*) and the corresponding information efficiency *I*_*E*_|_α = α(*i*)_, we evaluate the information efficiency when α = α(*L* + *i*) with *L* > 1, *I*_*E*_|_α = α(*L* + *i*)_. If *I*_*E*_|_α = α(*L*+*i*)_ > *I*_*E*_|_α = α(*i*)_, then evaluate *I*_*E*_|_α = α(2*L*+*i*)_, and so on. This process is repeated untill *I*_*E*_|_α = α(*QL*+*i*)_ < *I*_*E*_|_α = α((*Q*−1)*L*+*i*)_.

Backward phase: Starting from the point α(*QL* + *i*), which is obtained in the forward phase, we calculate *I*_*E*_|_α = α (*QL*+*i*−1)_. If *I*_*E*_|_α = α (*QL*+*i*−1)_ > *I*_*E*_|_α = α(*QL*+*i*)_, then calculate *I*_*E*_|_α = α(*QL*+*i*−2)_, and so on. This process is repeated until *I*_*E*_|_α = α (*QL*+*i*−*U*)_ < *I*_*E*_|_α = α(*QL*+*i*−*U*+1)_. We then get the optimum parameter α_*opt*_ = α(*QL* + *i* − *U* + 1) and the maximum information efficiency _*I*_*E*_*max*_ = *I*_*E*_|_α = α(*QL*+*i*−*U*+1)_.

For a firing rate distribution function with two free parameters, α_1_ and α_2_, another feature of the distribution function, the symmetry of the distribution function (see Equation 5), can be used to further reduce the computational complexity. Therefore, the algorithm of the search for the two parameters for the optimum information efficiency can be described as follows (the algorithm for a firing rate distribution function with three free parameters is similar to that with two free parameters).

Step 1: Discretize α_1_ and α_2_ using variable step size scheme discussed in the first paragraph in this subsection. We get discretized values, α_1_(1) = α_2_ (1) = 0.01, α_1_(2) = α_2_ (2) = 0.02,…, α_1_(4) = α_2_ (4) = 0.042,…, α_1_(65) = α_2_ (65) = 51 (we suppose that the values of the parameters are less than 51) for both α_1_ and α_2_.
Step 2: Let *i* = 1;
Step 3: Set α_1_ = α_1_(*i*);
Step 4: Set the initial value of α_2_ = α_2_(*i*) (note we do not set α_2_ = α_2_(1) due to the symmetry property);
Step 5: Use the forward and backward scheme for the parameter α_2_. Record the largest information efficiency and the corresponding parameter values of α_1_ and α_2_.
Step 6: Let *i* ← *i* + 1. If *i* ≤ 64 then goto Step 3.

### Searching the optimum neuronal firing rate distribution

**Figure 7:**
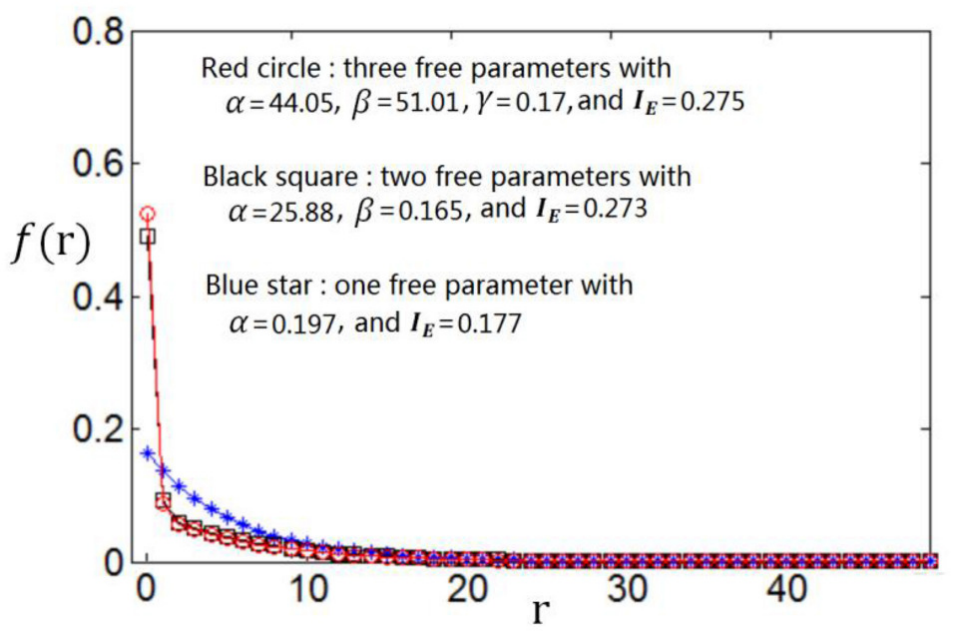
**Comparisons of the three kinds of firing rate distribution functions with different numbers of free parameters**. Equation (6) is used for the firing rate distribution with three free parameters. *E*_*b*_ = 2 and *c* = 1.

**Figure 8:**
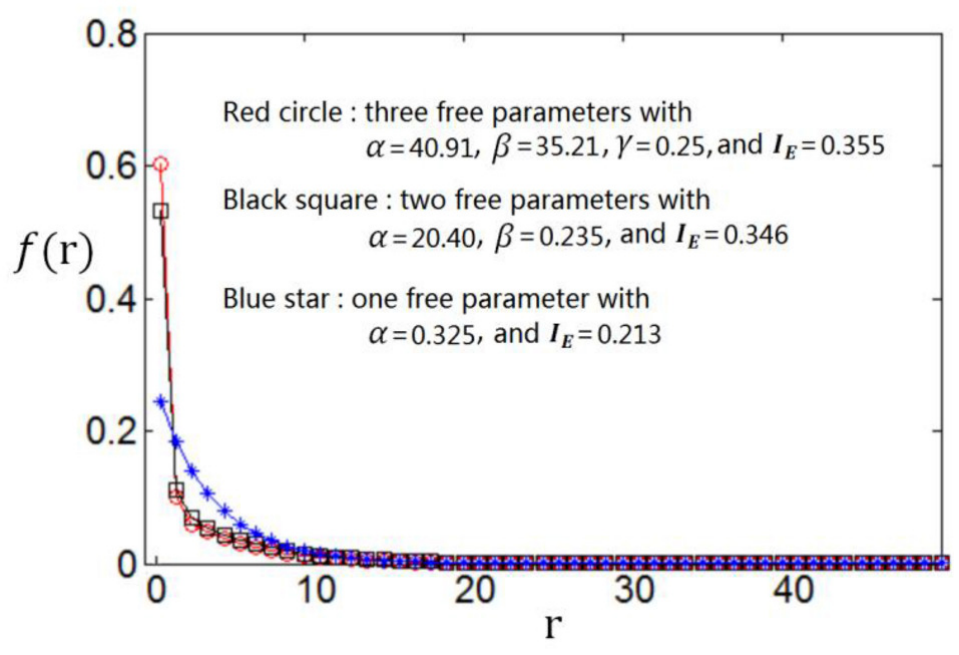
**Comparisons of the three kinds of firing rate distribution functions with different numbers of free parameters**. Equation (6) is used for the firing rate distribution with three free parameters. *E*_*b*_ = 1 and *c* = 1.

**Figure 9:**
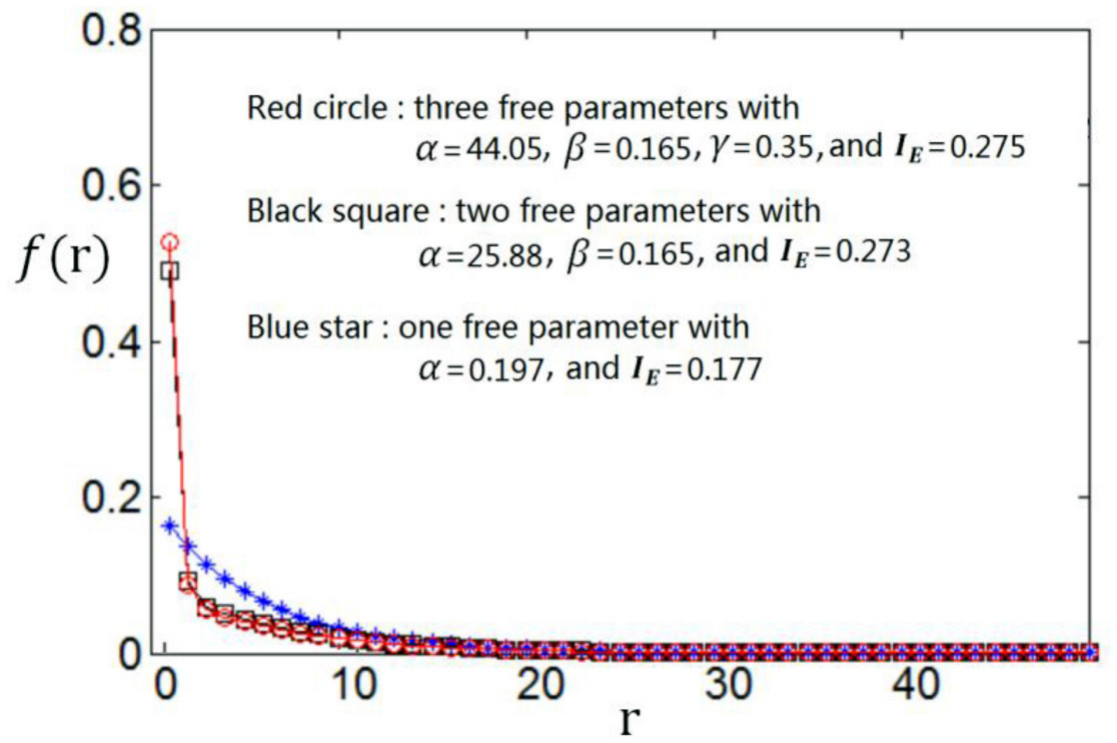
**Comparisons of the three kinds of firing rate distribution functions with different numbers of free parameters**. Equation (7) is used for the firing rate distribution with three free parameters. *E*_*b*_ = 2 and *c* = 1.

We use the proposed algorithm to search the optimum firing rate distribution for the maximum information efficiency. Among the four classes of firing rate distribution functions (Equations 4–7), we first determine which one is the best for searching the maximum information efficiency. It is found from Figures 7–**9** that the maximum information efficiency searched using firing rate distribution functions described by two free parameters is much higher than that using the functions described by only one free parameter. But the maximum information efficiency searched using firing rate distribution functions with three free parameters [see Figures 7, 8 where Equation (6) is used and Figure 9 where Equation (7) is used] is almost the same as that searched using the functions with two free parameters. Therefore, we believe that firing rate distribution functions described by two free parameters are good enough to describe the optimum firing rate distribution, and additionally they are of light computational complexity due to the few numbers of free parameters. Thus, we use the firing rate distribution of Equation (5) with two free parameters in the remainder of this paper.

**Figure 10:**
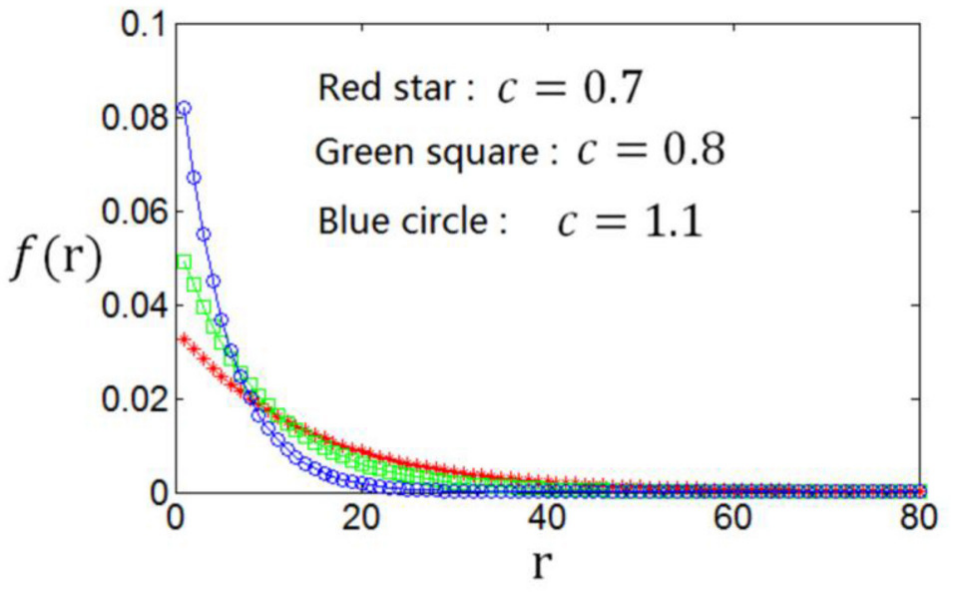
**Dependency of the optimum firing rate distribution on the parameter of the exponential index (***c***) of the information efficiency ***E***_***b***_ = 2**.

**Figure 11:**
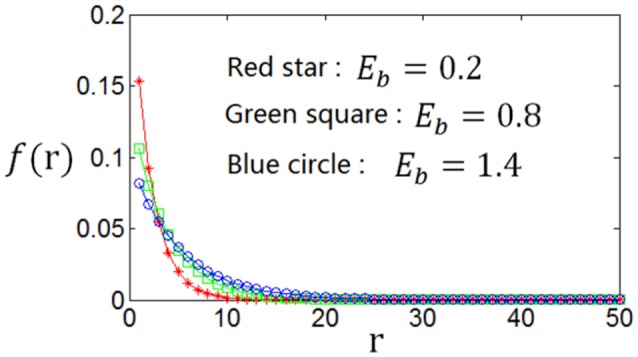
**Dependency of the optimum firing rate distribution on the parameter of the basic energy consumption (***E***_***b***_) of the information efficiency. ***c*** = 1**.

As the optimum firing rate distribution is different when a different definition of the information efficiency is used, we next explore the dependency of the optimum firing rate distribution on the parameters of the information efficiency. Figure 10 shows that when exponential index *c* becomes smaller, the optimum firing rate distribution will become flatter. This can be explained as follows. A firing rate distribution being relatively flat will result in large mutual information. Therefore, considering that small value of *c* means that energy consumption is not very important in evaluating the information efficiency, this relatively flat firing rate distribution will lead to maximum information efficiency, though this relatively flat distribution implies large amount of energy consumption [note $E_{s}=\sum_{n}P(n)n$]. Table 2 shows the dependency of the parameter values of the optimum firing rate distribution functions, α and β, on the parameter value of the information efficiency *c*. Figure 11 shows that if basic energy consumption of neurons (*E*_*b*_) is increased, the optimum firing rate distribution will become flat. This is not strange because large value of *E*_*b*_ means that the energy consumed by the neurons to emit spikes is relatively not important. Therefore, the optimum firing rate distribution is close to the one that leads to maximum mutual information, i.e., the optimum firing rate distribution is somehow flat. Table 3 shows the dependency of the parameter values of the optimum firing rate distribution functions, α and β, on the parameter values of the information efficiency *E*_*b*_.

**Table 2:** **Dependency of the values of α and β (parameters of the optimum firing rate) on the value of ***c*** (parameter of the information efficiency)**.

*c*	0.65	0.70	0.75	0.80	0.90	1.0	1.1	1.2
(α, β)	(0.06, 47)	(0.07, 41)	(0.09, 38)	(0.11, 33)	(0.14, 28)	(0.16, 26)	(0.20, 24)	(0.23, 22)
*I*_*E*_	0.546	0.484	0.434	0.392	0.324	0.273	0.232	0.198

**Table 3:** **Dependency of the values of α and β (parameters of the optimum firing rate) on the value of ***E***_***b***_ (parameter of the information efficiency)**.

*E*_*b*_	0	0.2	0.4	0.6	0.8	1.2	1.4	1.8
(α, β)	(0.98,20)	(0.51,19)	(0.38,20)	(0.32,20)	(0.28,20)	(0.23,22)	(0.20,22)	(0.16,24)
*I*_*E*_	0.633	0.504	0.442	0.400	0.370	0.326	0.310	0.283

## The tuning curves based on the optimum neuronal firing rate distribution

We can numerically compute the tuning curve *g*(·) (*r* = *g*(*s*)) easily if the probability distribution of the stimuli *s*, *P*(*s*(*i*)), and the distribution of the firing rate *r*, *P*(*r*(*j*)), are given. Let us make a summation of the probability of the stimuli, *P*(*s*(1)), *P*(*s*(2)), …, *P*(*s*(*M*)), one by one untill we get a number *d*_1_ (*d*_1_ > 1) such that $\overset{d_{1}}{\underset{i=1}{\sum }}P\left(s\left(i\right)\right)\leq P\left(r\left(1\right)\right)$ but $\overset{d_{1}+1}{\underset{i=1}{\sum }}P\left(s\left(i\right)\right)>P\left(r\left(1\right)\right)$. Then *r*(1) = *g*(*s*(*d*_1_)); similarly, we get *d*_2_ (*d*_2_ > *d*_1_) such that $\overset{d_{2}}{\underset{i=d_{1}+1}{\sum }}P\left(s\left(i\right)\right)\leq P\left(r\left(2\right)\right)$ but $\overset{d_{2}+1}{\underset{i=d_{1}+1}{\sum }}P\left(s\left(i\right)\right)>P\left(r\left(2\right)\right)$. Then *r*(2) = *g*(*s*(*d*_2_)); repeat this process and we obtain numerically the tuning curve *r*(*j*) = *g*(*s*(*d*_*j*_)), *j* = 1, 2, …, *M*.

**Figure 12:**
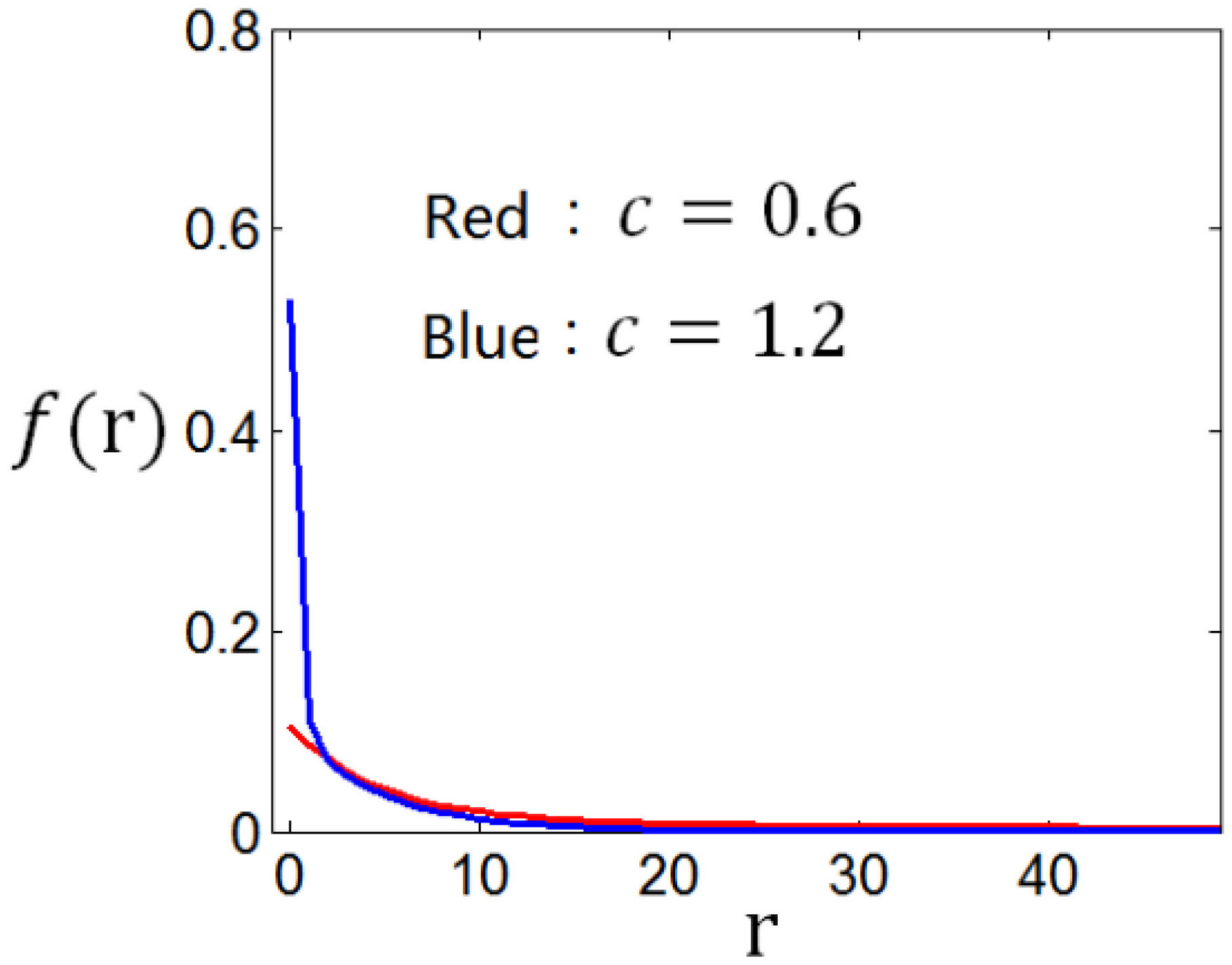
**Firing rate distributions used to calculate the neuronal tuning curves**.

**Figure 13:**
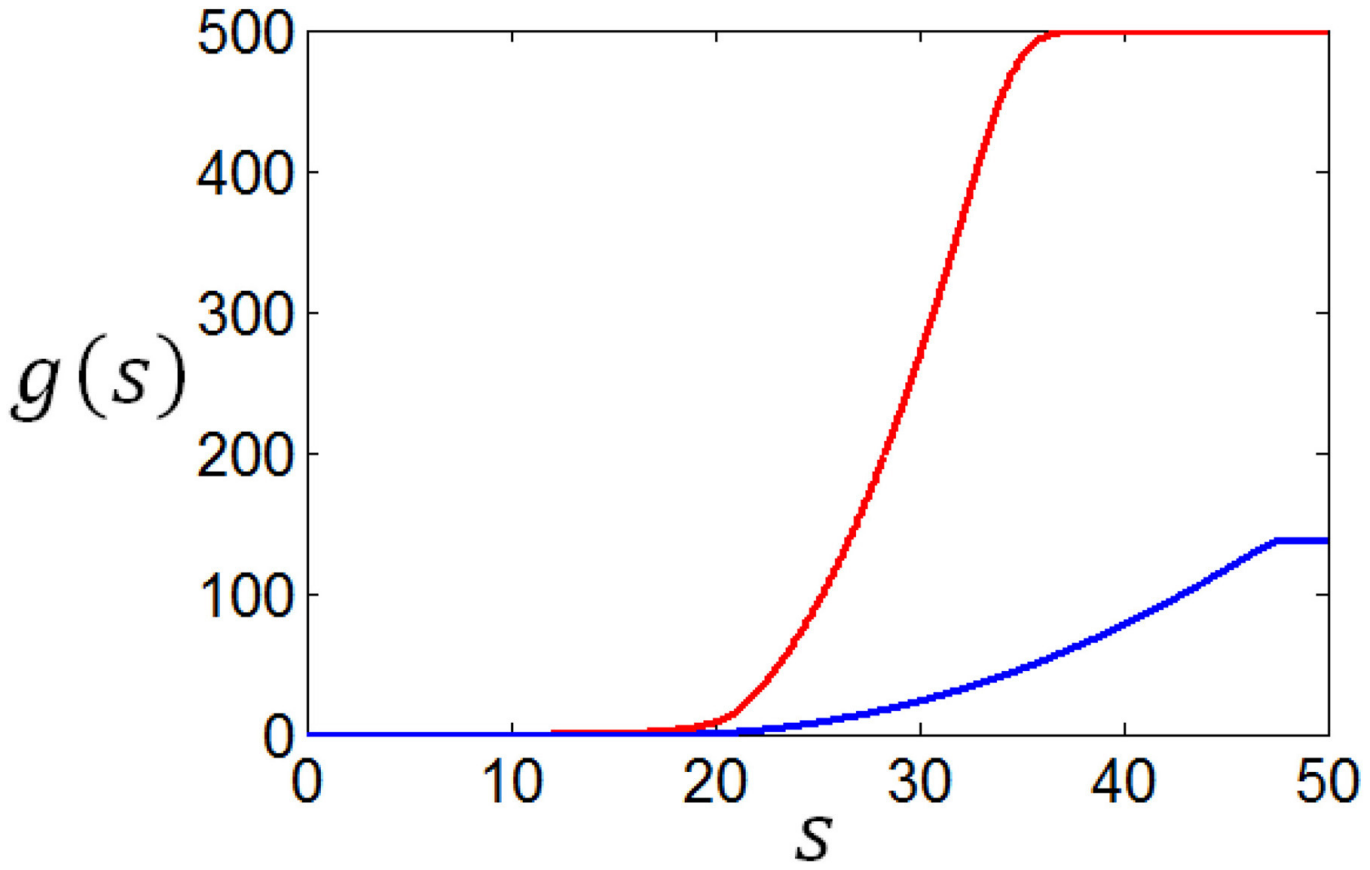
**The neuronal tuning curves corresponding to the firing rate distributions in Figure **12** and normal stimuli distribution**.

Using the method discussed in the previous paragraph, we calculate the tuning curves given different stimuli distributions and different firing rate distributions. Suppose the stimuli obey normal distribution, and the firing rate distributions are taken to be the two curves in Figure 12, we get corresponding two tuning curves in Figure 13. We can see from Figures 12, 13 that if energy consumption is not important compared to the mutual information (see the red curve in Figure 12, which is the optimum firing rate distribution with *c* = 0.6 in the definition of information efficiency), the tuning curve takes a sigmoid form (see red curve in Figure 13), which has been widely used in computational neuroscience. In the case where energy consumption is underlined (see the blue curve in Figure 12 which is the optimum firing rate distribution for the information efficiency with *c* = 1.2), the tuning curve (the blue one in Figure 13) take a form that it is below the one (the red one in Figure 13) that corresponding to the case when energy consumption matters little, and the top part of the sigmoid function is cut off.

**Figure 14:**
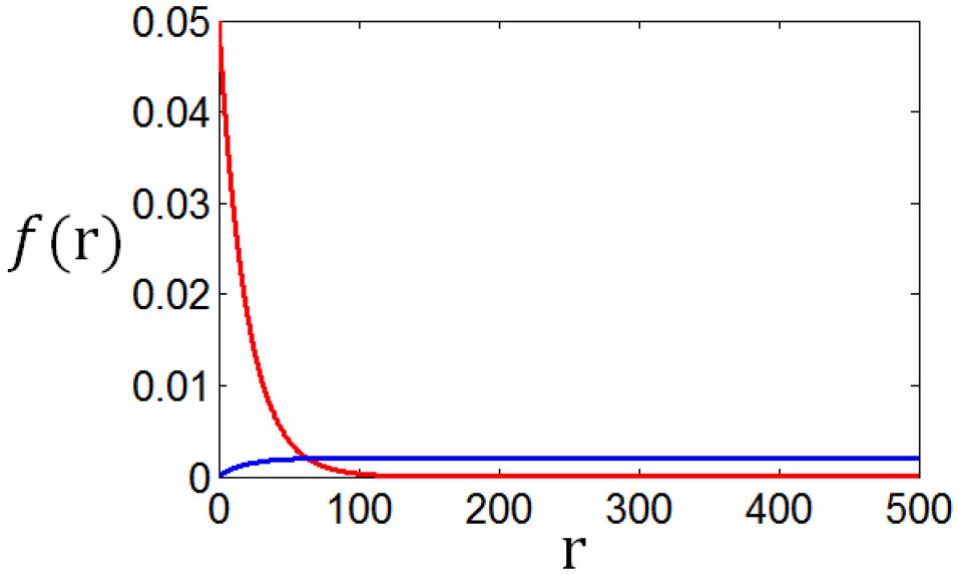
**Two arbitrary generated stimuli distributions**.

**Figure 15:**
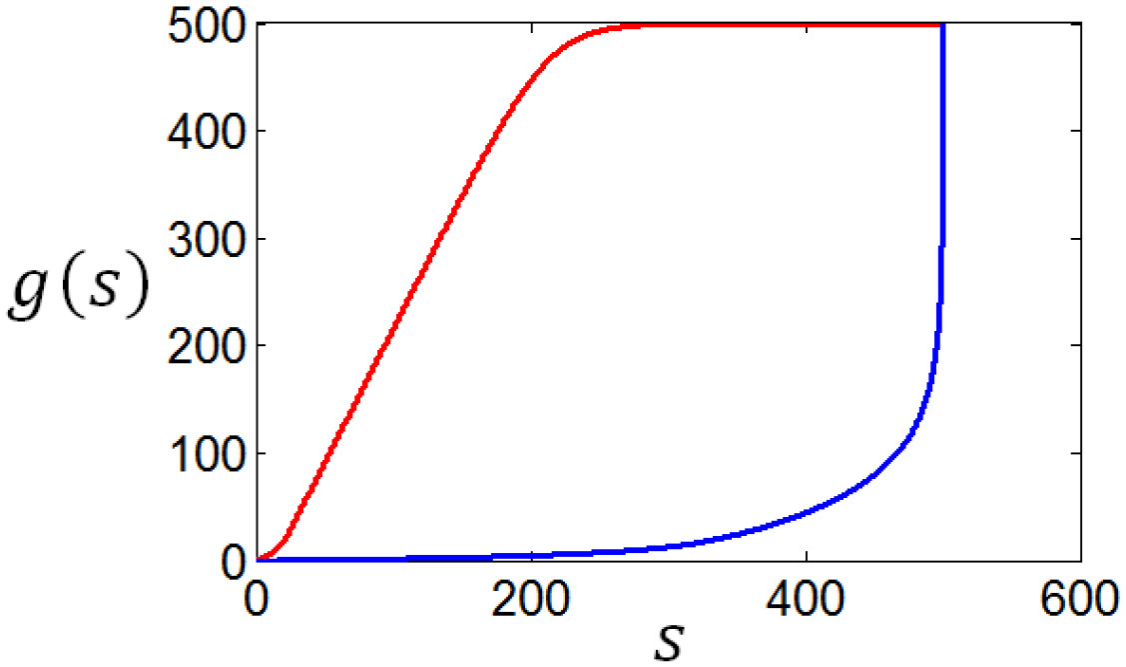
**The neuronal tuning curves corresponding to the red firing rate distribution in Figure **12** and the stimuli distributions in Figure **14****.

As a matter of fact, the tuning curves can be calculated for any stimuli distributions. Figure 14 shows two arbitrary curves of stimuli distribution, and the two curves in Figure 15 are the corresponding tuning curves.

## Conclusions

Neural systems are assumed to be optimized through biological evolution for millions of years for information processing, i.e., they are optimized to achieve highest information efficiency. Using this assumption, one can study many features of neuronal systems. The input–output relationships (tuning curves), *r* = *g*(*s*), have been explored in this paper. As one can calculate the tuning curve numerically if optimum firing rate distribution for information efficiency and the stimuli distribution are given, we focused on exploring the optimum firing rate distribution for the information efficiency in this paper. Firstly, a new definition for the information efficiency, $I_{E}=\left(2^{I_{m}}-1\right)/{\left(E_{s}+E_{b}\right)}^{c}$, has been given. The energy consumption consists of two components, the basic energy consumption *E*_*b*_ and the spike emission energy consumption *E*_*s*_. The relative importance of the energy consumption in the definition of the information efficiency is also modeled by the parameter *c*. Then, four main results concerning the optimum firing rate distribution have been obtained. (1) Contrast to the fact that the spike count response distribution should be flat for the maximum full entropy, the function of the spike count response distribution should decrease rapidly to 0 for the minimum spike emission energy consumption (*E*_*s*_). Therefore, the function of the optimum spike count response distribution for the entropy efficiency should exhibit a shape that decreases gradually to 0. This kind of functions can be described by a combination of the exponential functions. (2) Using the nonlinear least square method, we found that the optimum firing rate distribution function for the entropy efficiency has the same form of function as the optimum spike count response distribution. In other words, the function of the optimum firing rate distribution for the entropy efficiency can also be described by the combination of the exponential functions. Furthermore, we found that the dependency of the noise entropy on the shape of the firing rate distribution function is similar to that of the spike emission energy consumption, i.e., the function of the firing rate distribution should decrease rapidly to 0 to achieve the minimum noise entropy. Therefore, it can be concluded that a firing rate distribution function capable of producing high full entropy, low noise entropy and less energy consumption should have a shape decreasing gradually to 0, which can be described by a combination of exponential functions. (3) We developed a rapid algorithm with variable step size and forward-backward scheme to search the parameter values of the optimum firing rate distribution function. It has been found by this algorithm that firing rate distribution functions described by two free parameters, *f*(*r*) = (α*e*^−α*r*^ + β*e*^−β*r*^)/2, are accurate enough to describe the optimum firing rate distribution. Due to the small number of free parameters, they are of light computational complexity for the search for the optimum parameter values. (4) The dependency of the optimum firing rate distribution functions on the parameters of the information efficiency has been explored. It has been found that if exponential index *c* is decreased (the energy consumption is relatively neglected), the optimum firing rate distribution will become relatively flat. And if the basic energy consumption of neurons (*E*_*b*_) is increased, the optimum firing rate distribution will also become relatively flat. On the other hand, if exponential index *c* is increased (the energy consumption is relatively underlined) or *E*_*b*_ is decreased, the optimum firing rate distribution will become relatively steep. Finally, we designed an algorithm to calculate the tuning curves when the firing rate distribution and an arbitrary stimulus distribution are provided. By this algorithm, we found that in the case of normal stimuli distribution, the tuning curve exhibits a form of sigmoid if energy consumption is relatively neglected or the neuronal basic energy consumption is relatively large.

## Author contributions

All authors listed, have made substantial, direct and intellectual contribution to the work, and approved it for publication.

### Conflict of interest statement

The authors declare that the research was conducted in the absence of any commercial or financial relationships that could be construed as a potential conflict of interest.
